# Household headship and child death: Evidence from Nepal

**DOI:** 10.1186/1472-698X-10-13

**Published:** 2010-06-07

**Authors:** Ramesh Adhikari, Chai Podhisita

**Affiliations:** 1 Geography and Population Department, Mahendra Ratna Campus, Tribhuvan University, Kathmandu, Nepal; 2 Institute for Population and Social Research, Mahidol University, Salaya Campus, Nakhon Pathom, Thailand

## Abstract

**Background:**

Nepal has seen substantial improvements in its reproductive health outcomes, but infant and child mortality are still high. This study attempts to examine the prevalence and factors influencing the experience of child death of mothers who have given birth during a five-year period. More specifically, this paper aims to investigate whether household headship has an impact on child death in Nepal.

**Methods:**

This paper reports on data drawn from the Nepal Demographic and Health Survey (NDHS 2006), a nationally representative sample survey. The analysis is confined to women who had given birth during the five years preceding the survey (n = 4066). The association between experience of child death of mother and the explanatory variables was assessed via bivariate analysis using a chi-square test. The variables were also examined using multivariate analysis (binary logistic regression) to assess the net effect of household headship on child death after controlling for the other variables.

**Results:**

Out of all the mothers who had given birth during a five-years period, 3,229 (79.4%) were from male-headed households; the remaining 837 (20.6%) were from female-headed households. A significantly higher proportion of mothers from male-headed households (6.5%) than female-headed households (4.5%) had experienced the death of a child over the five years preceding the survey. Several socio-demographic, economic, and cultural variables were significant predicators for death of a child. For instance, women who had given birth to three or more children and who were Hindu were more likely to experience a child's death than were their counterparts. On the other hand, women who were literate, who had ever used family planning methods, who had visited a health facility, who utilized antenatal care for the last pregnancy, and who were from female-headed households were less likely to see a child die than were women in their comparison group. Notably, keeping all other control variables constant in the logistic model, women from female-headed households were 31 percent less likely to experience the death of a child (odds ratio = 0.69) than were women from male-headed households.

**Conclusion:**

The death of children is not uncommon in Nepal. No single factor can account for the high child mortality in the country; many factors contribute to the problem. After controlling for other variables, this study found that, among many other factors, household headship was a strong predictor. Programs seeking to help remedy this problem should focus on the issues identified here regarding women's autonomy, such as reducing the number of children born, increasing women's literacy status, increasing the use of family planning, increasing the use of antenatal care, and increasing female household headship so that child mortality will decrease and the overall well-being of the family can be maintained and enhance.

## Background

Considerable progress has been made to lower infant and child mortality rates throughout the world. Despite impressive achievements, each year approximately 10 million of the world's children under the age of five die from largely preventable diseases [1]. As in other developing countries, high infant and child mortality has been major public health problem in Nepal. Although over a past few decades, Nepal has seen substantial improvements in its reproductive health outcomes, infant and child mortality [2] as well as maternal mortality [3] are still very high compared to most other developing countries.

The basic social unit in Nepal is the family, consisting of a patrilineally extended household. Many extended families break apart as sons separate from parents. At the time of separation, the family property is equally divided among only the sons. Unmarried sons normally do not separate from their parents; if the parents are deceased, unmarried sons usually stay with their older married brothers. Women in Nepal are predominantly engaged in agricultural occupations and few have skilled manual jobs. They are less likely than men to be engaged in professional, technical, and managerial fields. On average a woman gives birth about three times (total fertility rate = 3.1). Most of these women spend their time doing domestic chores. Furthermore, a decade-long political insurgency (1995-2006) has resulted in a tremendous outflow of migrants, especially male members of households, to seek work in foreign countries, adding out-of-household responsibilities for the women left behind. Notably, more than a fifth of woman in Nepal still believe that a husband is justified in beating his wife in some situations [4].

In every aspect of life, women in Nepal are generally subordinate to men. Although the constitution offers women equal educational opportunities, many social, economic, and cultural factors contribute to lower enrollment and higher dropout rates for girls [5]. In many societies women's inferior status in terms of social status and status within the household adversely affects their health and that of their children. The health of women and their children are largely impaired by culturally and socially determined roles for women through a complex web of physiological and behavioral interrelationships and synergies that permeate every aspect of their lives [6]. Much research has shown that women's high status correlates positively with the health status of women and their children [7,8].

It is well documented that women almost everywhere are disadvantaged compared to men in terms of their access to assets, employment, health care, and education. Consequently, it is often assumed that female-headed households are poorer than male-headed households and are less able to invest in the education and health of children [9-12]. Many studies have focused on household headship, especially female-headed households and poverty [13-15]. However, very few studies have focused on household headship and reproductive health outcomes [16-18]. Since the status of Nepali women is low, with a concurrent low level of autonomy, their status in their households needs to be further explored in terms of health services utilization, which has a direct impact on maternal and child morbidity and mortality. This relationship clearly warrants further attention, particularly in settings such as Nepal, where maternal and child health utilization are low [19].

This study is an attempt to examine the prevalence and factors influencing the death of children. More specifically, this paper aims to investigate whether the sex of the head of the household has an impact on child mortality in Nepal. In addition, this paper also aims to fill the knowledge gap in the literature with regard to a society in which women suffer gross disadvantages by virtue of their roles and status in a patriarchal culture. The finding of this paper also helps reproductive health program planners and policy makers to understand the various factors influencing child mortality so as to implement reproductive health programs that will decrease infant and child mortality. Although a few studies on household headship and reproductive health outcomes do exist, this type of research has not yet been undertaken in Nepal.

## Methods

This paper reports on data drawn from the Nepal Demographic and Health Survey (NDHS, 2006), a nationally representative sample survey. The primary purpose of the 2006 NDHS was to furnish policymakers and planners with detailed information on fertility, family planning, mortality, maternal and child health, nutrition, and knowledge of HIV/AIDS and other sexually transmitted infections. The 2006 NDHS was carried out under the aegis of the Population Division of the Ministry of Health and Population.

The 2006 NDHS used the sampling frame provided by the list of census enumeration areas along with population and household information from the 2001 Population Census. Each of the 75 districts in Nepal were subdivided into Village Development Committees (VDCs) and each VDC into wards. The primary sampling unit (PSU) for the 2006 NDHS was a ward, subward, or group of wards in rural areas, and subwards in urban areas. The sample for the survey is based on a two-stage, stratified, nationally representative sample of households. At the first stage of sampling, 260 PSUs (82 in urban areas and 178 in rural areas) were selected using systematic sampling with probability proportional to size. At the second stage of sampling, systematic samples of about 30 households per PSU on average in urban areas and about 36 households per PSU on average in rural areas were selected in all the regions. Interviews were completed for 10,793 women of reproductive age [4].

The measurable outcome of the study is experience of child death of mothers who had given birth during the five-year period immediately preceding the survey, a dichotomous variable indicating whether or not the respondent had a child die within the past five years. The explanatory variables used in this study were based on the Mosley and Chen [20] determinant of childhood morbidity and mortality framework and other literature [21-23].

The unit of analysis in our study is women who had given birth during the five-year period (n = 4,066) immediately preceding the survey. Association between experience of child death of mothers and the explanatory variables was assessed via bivariate analysis using chi-square tests. Logistic regression was used to assess the net effect of household headship on child death after controlling for several other variables. Before the multivariate analysis, multicollinearity between the variables was assessed and the least important variables were removed from the logistic model.

## Results

Among the surveyed women (N = 10,793) of reproductive age, 7,809 women (72%) had previously given birth, and 4,066 (38%) had given birth during the five years preceding the survey. Among the women who had given birth during the five-year period, 3,229 (79.4%) resided in male-headed households; the remaining 837 women (20.6%) lived in female-headed households. Interestingly, among female-headed households, more than two-thirds of the mothers (68%) were themselves heads of their households. Furthermore, more than a fifth (22%) were daughters-in-law of the female head of the household in which they lived. A slightly higher proportion (43%) of women from male-headed households were youth aged 15-24 compared to the proportion of women from female-headed households (36%). It is notable that about a third (30%) of women had gotten married before the age of 16 in both male-and female-headed households. Among male-headed households, more than half of the women (52%) resided in the Terai region (the southern, flat strip of land bordering India). On the other hand, about half (49%). of the women who resided in the hills resided in female-headed households. It is encouraging to note that a significantly higher (p < 0.05) percentage of women (45%) from female-headed households were literate compared to women from male-headed households (41%). Significantly higher percentages (p < 0.001) of women from female-headed households (51%) were among the poor/poorest compared to those in male-headed households (43%) (table not shown).

More than one in twenty women (6%) who had given birth in the five years preceding the survey had experienced the death of a child. This percentage varied according to different socio-demographic, economic, and cultural settings. For instance, a significantly higher percentage (8%) of women who had gotten married at an early age (< 16 years) had lost a child, compared to women who had married at age 16 or later (5%). Similarly, the number of children ever born is positively associated with the number of children who died (4% for mothers who had had only one child to 8% for those who had had four or more children). A significantly higher percentage of women who had given birth within two years of a previous birth had experienced the death of a child (16%) than did women who had a two-year or more birth-spacing period (4%). Furthermore, urban women had a significantly lower experience of child death (4%) than did rural women (6%). Moreover, a significantly higher proportion (10%) of women who resided in Mountain regions had experienced the death of a child than had women in Hill (5%) or Terai (6%) regions (Table 1).

**Table 1:** Experience of child death of mother by socio-demographic characteristics (n = 4066).

Socio-demographic characteristics	Experience of child death within five years	
	
	Yes	No
**Age group**		
15-24 years	7.0	93.0
25-29 years	5.2	94.8
30-49 years	5.5	94.5

Age at first marriage **		
Less than 16 years	7.5	92.5
16 or more years	5.4	94.6

Number of children borne ***		
One	3.6	96.4
Two	5.5	94.5
Three	7.6	92.4
Four or more	8.0	92.0

Previous birth interval+ ***		
< 2 years	16.0	84.0
2 or more years	4.5	95.5

Place of residence **		
Urban	3.6	96.4
Rural	6.4	93.6

Ecological zone **		
Mountain	10.1	89.9
Hill	4.9	95.1
Terai	6.4	93.6

**Total**	**6.1**	**93.9**

Note *** Significant at P < 0.001; ** = p < 0.01 and * = p < 0.05; + = excludes first-order births.

Literacy is one factor that had a significant negative effect on children's death. Child mortality was lower among literate mothers (4%) than it was among illiterate mothers (8%). Similarly, a higher proportion of the poorest mothers (7%) had seen a child die compared to the richest mothers (5%). Those women who had never used any family planning methods had twice the experiences of children's deaths (9%) than did those who used family planning methods (4%). Interestingly, those who had visited a health facility in the 12 months preceding the survey for their own care or for the care of their children had a significantly lower experience of child death (5% vs. 8%). Moreover, only 5% of those women who had utilized a health service facility for antenatal care for their last birth had a child die compared to 9% of those who had not utilized such a facility. Notably, the percentage of women who had had a child die is significantly lower among those who resided in female-headed households (4.5%) than it was for women in male-headed households (6.5%) (Table 2).

**Table 2:** Experience of child death of mother by socioeconomic and cultural characteristics (n = 4,066).

Socioeconomic and cultural characteristics	Experience of child death within five years	
	
	Yes	No
Literacy status ***		
Illiterate	7.8	92.2
Literate	3.7	96.3

**Occupation**		
Not working/Agricultural sector	6.1	93.9
Non-agricultural sector	5.9	94.1

Religion *		
Non-Hindu	4.2	95.8
Hindu	6.4	93.6

**Mass media exposure**		
No exposure	6.3	93.7
One medium (Radio or TV) exposure	6.3	93.7
Both (Radio & TV) exposure	5.9	94.1

Wealth Status *		
Poorest	7.5	92.5
Poorer	5.5	94.5
Middle	7.6	92.4
Richer	4.5	95.5
Richest	4.6	95.4

Ever used of FP methods ***		
Never used	8.8	91.2
Ever used	4.4	95.6

Visited health facility for own or child's care in previous 12 months **		
No	7.9	92.1
Yes	5.2	94.8

ANC check for last birth ***		
No	8.5	91.5
Yes	5.2	94.8
Place of the last birth		
Home	6.4	93.6
Health facility	4.7	95.3

Household headship*		
Male-headed households	6.5	93.5
Female-headed households	4.5	95.5

**Total**	**6.1**	**93.9**

Note *** Significant at p < 0.001; ** = p < 0.01 and * = p < 0.05

While controlling for other control variables in the model, a binary logistic regression model was used to assess the net effect of independent variables (household headship) on the dependent variable (i.e., experience of child death). Crude and adjusted odds ratio are estimated and shown in Table 3. After assessing multicollinearity, it was found that the variables "age of woman" and "number of children ever born" were highly correlated. Therefore the variable "age of woman" was not entered into the logistic regression model.

**Table 3:** Crude and adjusted odds ratios (OR) for having experience of child death within the previous five years among women by selected predictors.

Predictors	Crude OR	Adjusted OR
**Age at first marriage **(Less than 16 years ref.)	1.00	1.00
16 or more years	0.70*	0.81

**Number of children borne **(One ref.)	1.00	1.00
Two	1.55*	1.02
Three	2.21***	171**
Four or more	2.33***	1.78**

**Previous birth interval **(< 2 years ref.)	1.00	1.00
2 or more years	0.24***	0.23***

**Place of residence **(Urban ref.)	1.00	1.00
Rural	1.82*	1.57

**Ecological zones **(Mountain ref.)	1.00	1.00
Hill	0.46***	0.58*
Terai	0.61*	0.67*

**Literacy status **(Illiterate ref.)	1.00	1.00
Literate	0.45***	0.56**

**Occupation **(Not working/agricultural sector ref.)	1.00	1.00
Non-agricultural sector	0.97	1.48

**Religion **(Non Hindu ref.)	1.00	1.00
Hindu	1.55*	1.79**

**Mass media exposure **(No exposure ref.)	1.00	1.00
One media (Radio or TV) exposure	1.00	1.32
Both (Radio & TV) exposure	0.95	1.58

**Wealth status **(Poorest ref.)	1.00	1.00
Poorer	0.72	0.88
Middle	1.01	1.35
Richer	0.58*	0.88
Richest	0.59*	1.50

**Ever use of FP methods **(Never used ref.)	1.00	1.00
Ever used	0.48***	0.43***

**Visited health facility for own or child's care in previous 12 months **(No ref.)	1.00	1.00
Yes	0.64**	0.73*

**ANC check for last birth **(No ref.)	1.00	1.00
Yes	0.59***	0.81*

**Place of the last birth **(Home ref.)	1.00	1.00
Health facility	0.71	1.15

**Household headship **(Male-headed ref.)	1.00	1.00
Female-headed	0.68*	0.69*

Note *** Significant at p < 0.001; ** = p < 0.01 and * = p < 0.05

In the model, several socio-demographic, economic, and cultural variables, such as number of children born, previous birth interval, ecological zone, literacy status, religion, use of family planning methods, visits to a health facility, antenatal care, and household headship, were significant predicators of children's deaths. Number of children born has a positive and statistically significant impact. For example, women who had given birth three or more times were about two times more likely to experience the death of a child compared to those who had given birth only once. On the other hand, women who had longer birth spacing (two years or more) were 77% less likely (odds ratio = 0.23) to have a child die than were those who had shorter birth spacing (< two years). Similarly, those women who lived in the Hill and Terai regions were less likely (odds ratio, Hill = 0.58, Terai = 0.67) to experience a child's death than were those who lived in the Mountain region. Literate women were 44% less likely (odds ratio = 0.56) to have a child die than were illiterate women. Furthermore, Hindu women were more likely to have a child die (odds ratio = 1.79) than were women who followed other religions. Those women who had used family planning methods were 57% less likely to experience the death of a child (odds ratio = 0.43) than were those who had never used family planning. Similarly, women who had visited a health facility for their own or their children's care in the 12 months preceding the survey were 27% less likely to experience a child's death (odds ratio = 0.73) than were women who had not visited a health facility during that period. Women who had utilized a health facility for antenatal care during their last pregnancy were 19% less likely to have a child die (odds ratio = 0.81) than were those who had not utilized such a service. Furthermore women in female-headed households were 31% less likely to experience the death of a child (odds ratio = 0.69) than were women in male-headed households, keeping all other variables constant in the model (Table 3).

## Discussion

This study has attempted to investigate the influence of particular sociodemographic, economic, and cultural factors--especially household headship--on child deaths in Nepal. The present study shows that the experience of child death is not uncommon among Nepalese women and indicates an unmet need for an effective reproductive health program.

The bivariate analysis shows that variables such as age at first marriage, children ever born, place of residence, ecological zone, literacy status, religion, wealth status, use of family planning methods, visits to a health facility, antenatal care for the last pregnancy, and household headship are important in explaining child mortality. The multivariate analysis supports many of the findings of the bivariate analysis. In the multivariate analysis, the number of children born, ecological zone, literacy status, religion, use of family planning methods, visits to a health facility, antenatal care for last pregnancy, and household headship are found to have a statistically significant influence on child mortality.

As have many other studies, this study shows a strong relationship between women's fertility behavior and child mortality. Those women who had given birth to more children were more likely to have a child die. Moreover, our study also found that women who had short birth spacings were more likley to have a child die. Short birth spacing and high birth order may affect both maternal and fetal health as well as diminishing the time available for child care. This finding conforms with a study by Antonoyosky and Bernstein indicating that high parity women have the highest risk of child mortality [24].

This study found that women in the Hills and the Terai regions had a lower experience of child death than did women who resides in the mountains. Access to transportation and health care facilities in the Hill and Terai regions is much greater than it is in the Mountain region. Similarly, a higher proporiton of the urban population lives in Terai and Hill regions than in the Mountain region. The urban-rural differentials may be attributed to different health care services, including higher immunization coverage, safer childbirth, and better access to health care[25].

Similar to many other studies, this study shows that literate mothers a group see fewer of their children die. Educated mothers tend to be less fatalistic about their children's illnesses, are more capable of gaining access to available health facilities, and greatly alter the traditional balance within familial relationships, with profound effects on child care [21]. Furthermore, educated mothers are more likley to have received antenatal care [21,26]. Another reason could be that educated women are better able to utilize what is available in the community to their advantage [27,28]. Moreover, education can also contribute to children's survival by helping delay marriage and motherhood, decreasing the total number of children a woman has [18], and encouraging prenatal care and the immunization of children [29].

This study found that Hindu women were more likley to have a child die than were women of other religions. This could be due to cultural beliefs and practices that often lead to self-care, home remedies, and consulation with traditional healers [30]. These factors result in a delay in seeking appropriate treatment and are more common among women, not only with regard to their own health but especially that of their children [31,32].

Our study found that women who had used family planning methods were less likely to experience the death of a child than were those who had never used them. Family planning methods help lengthens the time between births. This finding agrees with many others that find that the length of the birth interval is positively correlated with the survival of the child [22,33,34]. Similarly, our study shows that those who had visited a health facility for their own health or their children's care in the 12 months preceding the survey had, as a group, fewer children die. This could be due to the fact that educated mothers are more likely to recognize a health problem and thus seek medical care [35]. The other reason could be that these women were more aware of family planning methods due to the many family planning posters/pamphlets that can be seen in health facilities, and so they might have been motivated to use these methods. The lower child mortality rate might also result from visits to health facilities that made mothers aware of the immunization requirements of their children. Similarly, our study also found that women who had utilized antenatal services for their last pregnancy had fewer children die than did those who did not. Antenatal visits can play a critical role in preparing a women and her family for birth by establishing rapport and a feeling of confidence between the women and the healthcare provider and by individualizing promotional health messages [36]. Antenatal care provides an opportunity for a variety of preventive interventions during pregnancy, including tetanus toxoid injections, and educating women about nutrition, safe delivery, and postpartum care [37].

Our study shows that women from female-headed households were less likely to experience the death of a child than were women from male-headed households. This could be due to the fact that women could talk more easily with the female head of the household about their reproductive health problem as well as children's illnesses. The other reason could be that female household heads could better understand female health problems, so they encouraged women to visit health facilities to treat a sick child when necessary. Interestingly, more than two-thirds of the women from female-headed households were themselves heads of their households. A possible explanation for these women's lower experience of children's death could be that women who have autonomy in decision making are more likely to use contraceptives, which might decrease the risks associated with reproductive behavior, prolong birth intervals, lessen their fertility [23], and ultimately minimize child mortality [22,33,34]. Research in India has confirmed that a woman's control over household resources (ability to keep money aside) has a significant positive effect on both the demand for prenatal care and the probability of hospital delivery [38]. A similar study in Sri Lanka found that women in female-headed households used health services more frequently than did those in male-headed ones [16]. Since the study of household headship is likely to be context specific, a qualitative approach may provide more insight to fully explore the further relationship between household headship and child mortality in Nepal.

There are some limitations in the interpretation of the results of this study. First, as pointed out previously, we restricted our subjects to only those women who had given birth within the five years preceding the survey, so our results regarding the prevalence of the experience of child death should not be generalized to all women in Nepal. Second, because the cross-sectional design of the study and all of the items analyzed in the logistic regression analysis came from information at the time of survey, the analysis can only provide evidence of statistical association between those items and the child death and cannot show cause-effect relationships.

## Conclusion

Child death is not uncommon in Nepal. No single factor can account for the high child mortality in the country; many factors contribute to the problem. After controlling for other variables, this study found that, among many other factors, household headship was a strong predictor. Programs seeking to help remedy this problem should focus on the issues identified here regarding women's autonomy, such as reducing the number of children born, increasing women's literacy status, increasing the use of family planning, increasing the use of antenatal care, and increasing female household headship so that child mortality will decrease and the overall well-being of the family can be maintained and enhance.

## Competing interests

The authors declare that they have no competing interests.

## Authors' contributions

RA analyzed and interpreted the data and drafted the manuscript. CP commented on the analysis and interpretation and was involved in drafting the manuscript. Both authors read and approved the final version of the manuscript.

## Pre-publication history

The pre-publication history for this paper can be accessed here:

http://www.biomedcentral.com/1472-698X/10/13/prepub
